# Supplementary material for: Causal Relationship between Aging and Anorexia Nervosa: A White-Matter-Microstructure-Mediated Mendelian Randomization Analysis
Source: Biomedicines. 2024 Aug 16;12(8):1874. doi: 10.3390/biomedicines12081874 (PMC11351342; doi:10.3390/biomedicines12081874)
Supplement: Supplementary file 1 [file biomedicines-12-01874-s001.zip › Supplementary Figure/Figure S4/Singe SNP plot of MR analysis for TL to ubm-b-1468.pdf]

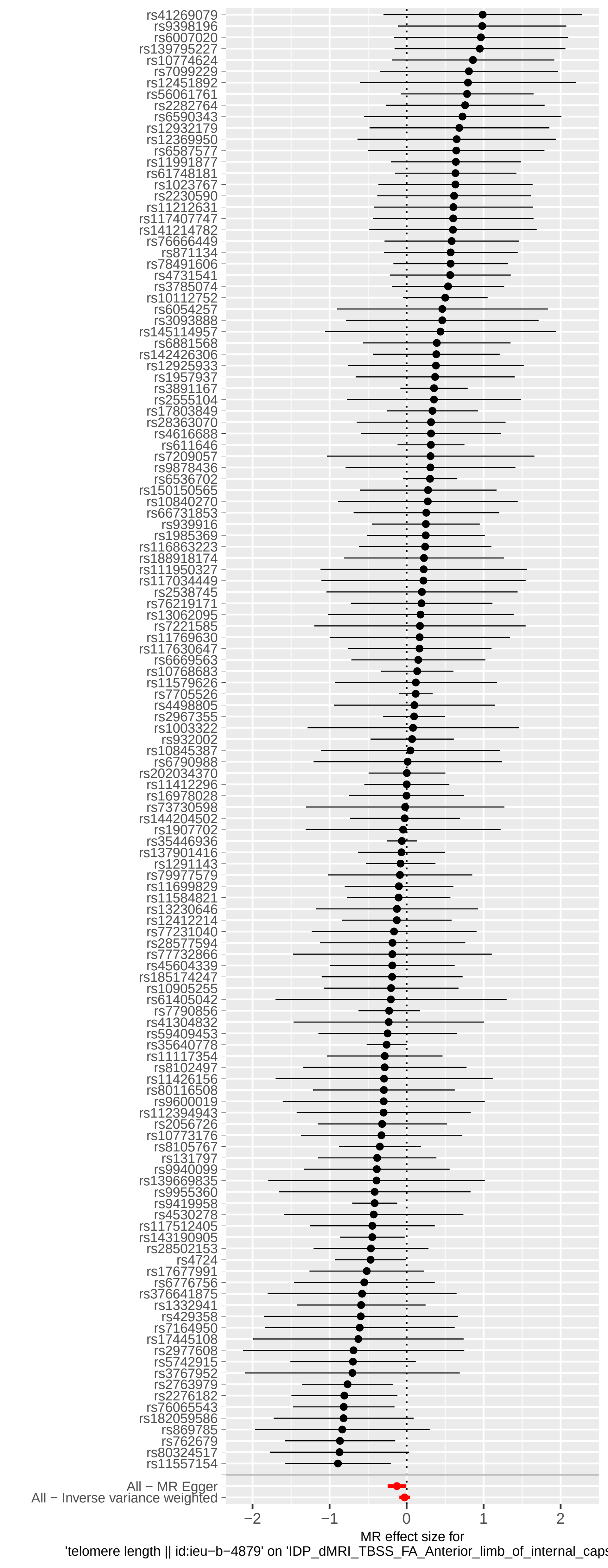
rs41269079
rs9398196
rs6007020
rs139795227
rs10774624
rs7099229
rs12451892
rs56061761
rs2282764
rs6590343
rs12932179
rs12369950
rs6587577
rs11991877
rs61748181
rs1023767
rs2230590
rs11212631
rs117407747
rs141214782
rs76666449
rs871134
rs78491606
rs4731541
rs3785074
rs10112752
rs6054257
rs3093888
rs145114957
rs6881568
rs142426306
rs12925933
rs1957937
rs3891167
rs2555104
rs17803849
rs28363070
rs4616688
rs611646
rs7209057
rs9878436
rs6536702
rs150150565
rs10840270
rs66731853
rs939916
rs1985369
rs116863223
rs188918174
rs111950327
rs117034449
rs2538745
rs76219171
rs13062095
rs7221585
rs11769630
rs117630647
rs6669563
rs10768683
rs11579626
rs7705526
rs4498805
rs2967355
rs1003322
rs932002
rs10845387
rs6790988
rs202034370
rs11412296
rs16978028
rs73730598
rs144204502
rs1907702
rs35446936
rs137901416
rs1291143
rs79977579
rs11699829
rs11584821
rs13230646
rs12412214
rs77231040
rs28577594
rs77732866
rs45604339
rs185174247
rs10905255
rs61405042
rs7790856
rs41304832
rs59409453
rs35640778
rs11117354
rs8102497
rs11426156
rs80116508
rs9600019
rs112394943
rs2056726
rs10773176
rs8105767
rs131797
rs9940099
rs139669835
rs9955360
rs9419958
rs4530278
rs117512405
rs143190905
rs28502153
rs4724
rs17677991
rs6776756
rs376641875
rs1332941
rs429358
rs7164950
rs17445108
rs2977608
rs5742915
rs3767952
rs2763979
rs2276182
rs76065543
rs182059586
rs869785
rs762679
rs80324517
rs11557154
All − MR Egger
All − Inverse variance weighted
−2
−1
0
1
2
MR effect size for
'telomere length || id:ieu−b−4879' on 'IDP_dMRI_TBSS_FA_Anterior_limb_of_internal_caps
